# Biocatalysis of triglycerides transesterification using fungal biomass: a biorefinery approach

**DOI:** 10.1186/s40694-023-00160-3

**Published:** 2023-06-12

**Authors:** Nadeem I. Elhussiny, Ahmed M. A. Mohamed, Heba A. El-Refai, Sayeda S. Mohamed, Yousseria M. Shetaia, Hala A. Amin, Gerd Klöck

**Affiliations:** 1 Department of Life Science and Chemistry, Constructor University, Bremen Campus Ring 1, 28759 Bremen, Germany; 2 Department of Chemistry of Natural and Microbial Products, National Research Centre, Dokki, Cairo, 12622 Egypt; 3 Institute of Environmental Biology and Biotechnology, University of Applied Sciences, 28199 Bremen, Germany; 4 Department of Botany and Microbiology, Faculty of Science, Helwan University, 11795 Cairo, Egypt; 5 Department of Microbiology, Faculty of Science, Ain Shams University, Abbassia, 11566 Cairo, Egypt

**Keywords:** Biorefinery, Transesterification, *Aspergillus flavus*, *Rhizopus stolonifer*, Wastewater, Fungal biomass, Biocatalysis

## Abstract

**Background:**

The use of microbial biomasses, such as fungal biomass, to catalyze the transesterification of triglycerides (TG) for biodiesel production provides a sustainable, economical alternative while still having the main advantages of expensive immobilized enzymes.

**Results:**

Biomasses of *Aspergillus flavus* and *Rhizopus stolonifera* were used to catalyze the transesterification of TG in waste frying oil (WFO). Isopropanol as an acyl-acceptor reduced the catalytic capability of the biomasses, while methanol was the most potent acyl-acceptor with a final fatty acid methyl ester (FAME) concentration of 85.5 and 89.7%, w/w, for *R. stolonifer* and *A. flavus*, respectively. Different mixtures of the fungal biomasses were tested, and higher proportions of *A. flavus* biomass improved the mixture's catalytic capability. *C. sorokiniana* cultivated in synthetic wastewater was used as feedstock to cultivate *A. flavus.* The biomass produced had the same catalytic capability as the biomass produced in the control culture medium. Response surface methodology (RSM) was adopted using central composite design (CCD) to optimize the *A. flavus* biomass catalytic transesterification reaction, where temperature, methanol concentration, and biomass concentration were selected for optimization. The significance of the model was verified, and the suggested optimum reaction conditions were 25.5 °C, 250 RPM agitation with 14%, w/w, biomass, 3 mol/L methanol, and a reaction duration of 24 h. The suggested optimum conditions were tested to validate the model and a final FAME concentration of 95.53%. w/w was detected.

**Conclusion:**

Biomasses cocktails might be a legitimate possibility to provide a cheaper technical solution for industrial applications than immobilized enzymes. The use of fungal biomass cultivated on the microalgae recovered from wastewater treatment for the catalysis of transesterification reaction provides an additional piece of the puzzle of biorefinery. Optimizing the transesterification reaction led to a valid prediction model with a final FAME concentration of 95.53%, w/w.

## Background

**Fig. 1:**
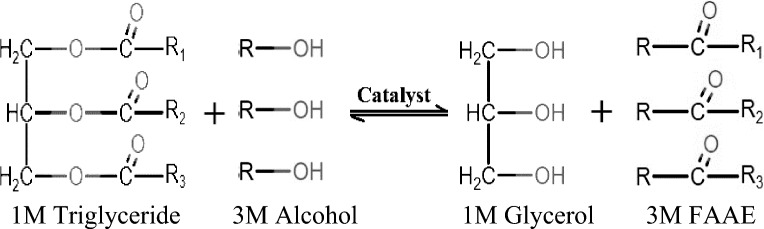
Triglycerides transesterification reaction for FAAE production

Low volatility, high viscosity, and polyunsaturated criteria are the primary disadvantages of using crude vegetable oils as diesel. Several methods were employed to overcome these problems, including blending with hydrocarbons [1], pyrolysis [2], micro-emulsions preparation[3], and transesterification [4]. The industrial production of biodiesel relies mainly on the transesterification of vegetable oils [5]. Triglycerides react with alcohol to produce fatty acid alkyl ester (FAAE) in a catalytic reaction, as shown in Fig. 1.

Catalyst improves the transesterification reaction rate and increases the yield of the FAAE. The catalysts used in biodiesel production are classified as homogeneous, heterogeneous, and biocatalysts. Employing a homogenous catalyst (acid or base) was not applicable on a large scale due to the poor quality of the produced biodiesel, which requires multiple washing and purification steps to meet the quality, hence increasing the cost, in addition to the risk of saponification of the oil [6]. On the other hand, heterogeneous catalysis demands high energy and pressure inputs and a free fatty acid (FFA) free feedstock [7].

Biocatalysts provide an eco-friendly option that usually catalyze the transesterification reaction under mild operation conditions and facilitate product separation without any byproduct formation. Employing enzymes for the catalysis transesterification reactions face critical problems, such as the cost and stability of enzymes. The use of microbial biomasses to catalyze transesterification, such as fungal biomasses, has been gaining research interest [8, 9] because it provides a potential cost-competitive option while still having the main advantages of immobilized enzymes that include reusability, mild reaction conditions, the capability of catalyzing glycerides and free fatty acids (FFA), no risk of saponification, producing high degree glycerol as a side reaction, short reaction time, and being an environmentally friendly option [10].

Microalgae biorefineries could efficiently fulfill a considerable part of the increasing fuel demand and reduce greenhouse gases, directly interacting with global warming and climate change. However, developing a biorefinery resembles the mosaic assembly that requires gathering numerous sustainable approaches and techniques and connecting them to build an overview of the whole process. In this regard, a biorefinery approach was assessed, in this study, by examining the ability of fungal biomass produced by cultivation in a culture medium of microalgae feedstock collected from wastewater treatment was examined, and the transesterification reaction conditions were optimized to maximize the transesterification capability of the fungal biomass. In addition, the capability of biomass cocktails from different fungal species to catalyze the transesterification reaction was evaluated.

## Results

### One factor at a time (OFAT) investigations

*Aspergillus flavus* and *Rhizopus stolonifer* were cultivated in a control medium under submerged conditions, and the produced biomass was harvested by filtration. Dried biomass was used as a catalyst for the transesterification reactions carried out in this section.

**Fig. 2:**
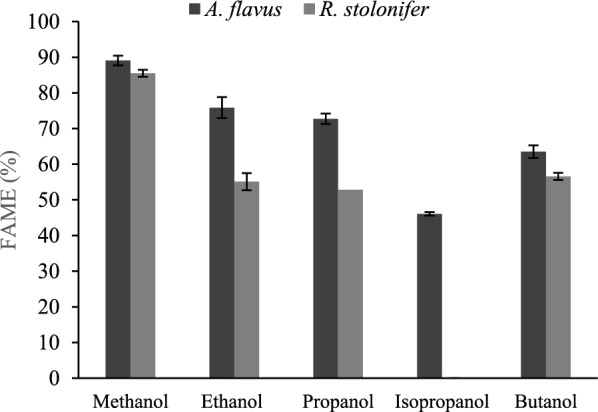
Effect of different alcohols on the transesterifications catalytic capability of *Aspergillus flavus* and *Rhizopus stolonifera* biomasses

Different short-chain alcohols were tested as acyl-acceptors for the transesterification of TG in WFO. Stepwise addition of alcohol was employed where alcohols were added in 3 doses separated within 24 h. Results (Fig. 2) showed that under the investigated reaction conditions, isopropanol as an acyl-acceptor inhibited the transesterification of TG, where *R. stolonifer* biomass was used as the catalyst, while a relatively low FAAE concentration was achieved where *A. flavus* biomass was employed. FAAE maximum concentration was detected in reactions where methanol was used as the acyl-acceptor, and it showed a significant difference between *A. flavus* and *R. stolonifer* biomasses, 89.09 and 85.50, respectively. Hence methanol was selected for further investigations.

**Fig. 3:**
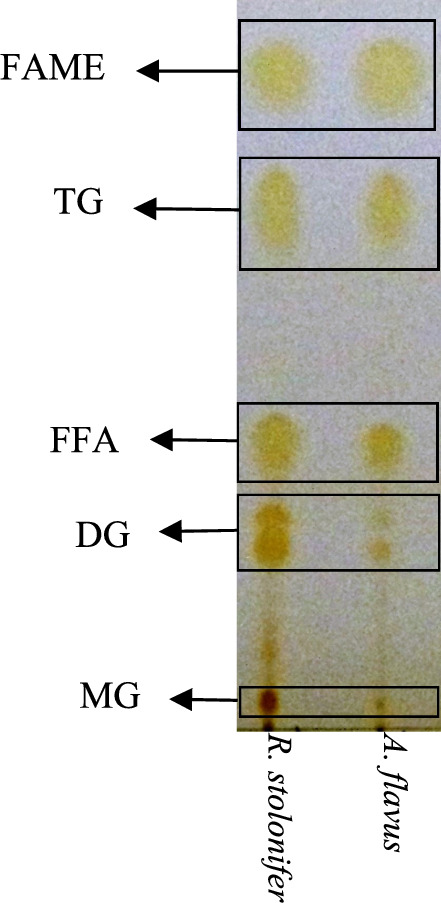
Thin layer chromatography separation pattern of transesterification reaction products and intermediates

TLC separation pattern (Fig. 3) of the transesterification reaction products revealed a difference in the accumulated intermediates during the reaction and over the reaction time between reactions catalyzed by *A. flavus* and *R. stolonifer* biomasses. In the reaction catalyzed by *R. stolonifer* biomass, the accumulation of the fatty acid methyl ester (FAME) was accompanied by the accumulation of free fatty acids (FFA), diglycerides (DG), and monoglycerides (MG). On the other hand, in the reaction catalyzed by *A. flavus* biomass, the accumulation of FFA, DG, and MG was minimal. These observations could only be understood in light of the reaction mechanism. Still, because biomass is used, only the dominant reaction mechanism could be discussed without excluding the presence of other mechanisms.

Different biomass mixtures (Table 1) were tested as catalysts for the transesterification of TG in WFO, FAME concentration by the end of the reaction time was 84.43–90.07%, w/w. Mixtures A and B were just pure biomasses as reference values, revealing that *A. flavus* biomass catalytic capability was significantly superior to *R. stolonifera* biomass. None of the tested mixtures showed a transesterification capability higher than mixture B which was the pure *A. flavus* biomass.

**Table 1:** Biomass mixtures for transesterification catalysis

Mixture	*A. flavus*	:	*R. stolonifer*	FAME %	
A	0	:	1	85.50	bc
B	1	:	0	89.69	a
C	1	:	1	88.74	ab
D	1	:	2	87.65	b
E	1	:	3	84.43	c
F	2	:	1	89.50	a
G	3	:	1	90.07	a

Means followed by the same letter are not significantly different at a confidence level of 95%

### Biorefinery approach

*Chlorella sorokiniana* was cultivated in synthetic wastewater and produced microalgae biomass was used as a feedstock for the cultivation of *A. flavus*. The final concentration of *C. sorokiniana* biomass was 3.5 gL^−1^ after eight days of 500 mL cultures incubation in 1L Erlenmeyer flasks, daylight, and room temperature. Algal biomass was harvested by centrifugation, and the harvested biomass was used as feedstock. The growth of *A. flavus* and its biomass transesterification capability was tested at different concentrations of *C*. *sorokiniana* feedstock. Results (Table 2) show that *A. flavus* could grow in culture media containing only *C. sorokiniana* biomass and olive oil. Although the final *A. flavus* concentration was low compared to the control, the yield in terms of conversion is higher in the *C. sorokiniana* biomass-containing medium. On the other hand, no significant difference was observed in the lipase activity or the transesterification capability of the produced *A. flavus* biomass, which indicates that the produced biomass was efficient as a catalyst for tested reactions.

**Table 2:** Growth and catalytic capability of *A. flavus* produced in media containing different algal feedstock concentration

Medium	Growth (gL^−1^)		Lipase (Ug^−1^)		FAME (%, w/w)	
*C. sorokiniana* biomass (%, w/v)						
0.25	2.18	d	18.28	a	89.30	a
0.50	4.12	c	18.17	a	90.12	a
1.00	7.93	b	18.22	a	90.00	a
Control	19.07	a	18.05	a	89.74	a

Means followed by the same letter are not significantly different at a confidence level of 95%

### Reaction optimization:

Response surface methodology (RSM), using a central composite design (CCD), was adopted to determine the optimum levels of the selected factors (temperature, methanol concentration, and biomass concentration), where FAME concentration was defined as the response. The design matrix and the corresponding results of CCD experiments to assess the effects of the three investigated factors are shown in Table 3. The prediction formula was simplified to a second-order polynomial equation. The response, FAME (Y), can be expressed in terms of the following regression equation:

$${\text{Y }} = { 26}.{74 } + { 1}.{\text{66X}}_{{1}} + { 9}.{\text{26X}}_{{2}} + { 58}.{\text{88X}}_{{3}} - \, 0.0{\text{5X}}_{{1}}^{{\mathbf{2}}} + \, 0.{\text{34X}}_{{2}}^{{\mathbf{2}}} - { 15}.{4}0{\text{X}}_{{3}}^{{\mathbf{2}}} - \, 0.{4}0{\text{X}}_{{1}} {\text{X}}_{{2}} + { 2}.{\text{67X}}_{{1}} {\text{X}}_{{3}} - { 11}.{\text{24X}}_{{2}} {\text{X}}_{{3}}$$

**Table 3:** Central Composite Design matrix and experimental results

Runs	X_1_	X_2_	X_3_	FAME (%, w/w) The mean of 2 trials
01, 02	− 1	− 1	− 1	56.36
03, 04	− 1	− 1	1	91.45
05, 06	− 1	0	0	78.15
07, 08	− 1	1	− 1	68.44
09, 10	− 1	1	1	88.81
11, 12	0	− 1	0	78.31
13, 14	0	0	− 1	42.22
15, 16	0	0	0	58.67
17, 18	0	0	1	78.57
19, 20	0	1	0	47.96
21, 22	1	− 1	− 1	10.31
23, 24	1	− 1	1	89.73
25, 26	1	0	0	36.23
27, 28	1	1	− 1	2.90
29, 30	1	1	1	43.06

**Fig. 4:**
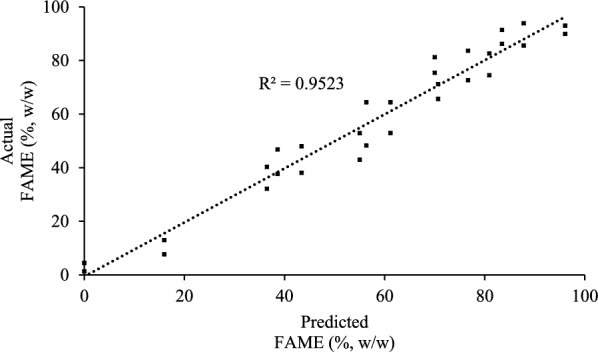
Central composite design, actual VS predicted FAME concentration values

The model was verified via the results of the analysis of variance, ANOVA (Table 4). The model was significant, with p-values below 0.0001. The model's lack of fit was insignificant, with p-values higher than 0.05. Plotting the actual values obtained from the experiments against the predicted values deducted by the model (Fig. 4) supports the findings of the adequacy of the models, where R^2^ values were 0.95.

**Table 4:** Analysis of variance (ANOVA) for the parameters of response surface methodology fitted to a second-order polynomial equation

Source	DF	SS	MS	F-Value	P-value > F
*Model*	9	21,063.10	2340.34	43.44	< 0.001
*Error (Residual)*	20	1077.44	53.87		
*Lack of Fit*	5	510.13	102.03	2.70	0.06228
*Pure Error*	15	567.31			
*Total*	29	22,140.54

**Fig. 5:**
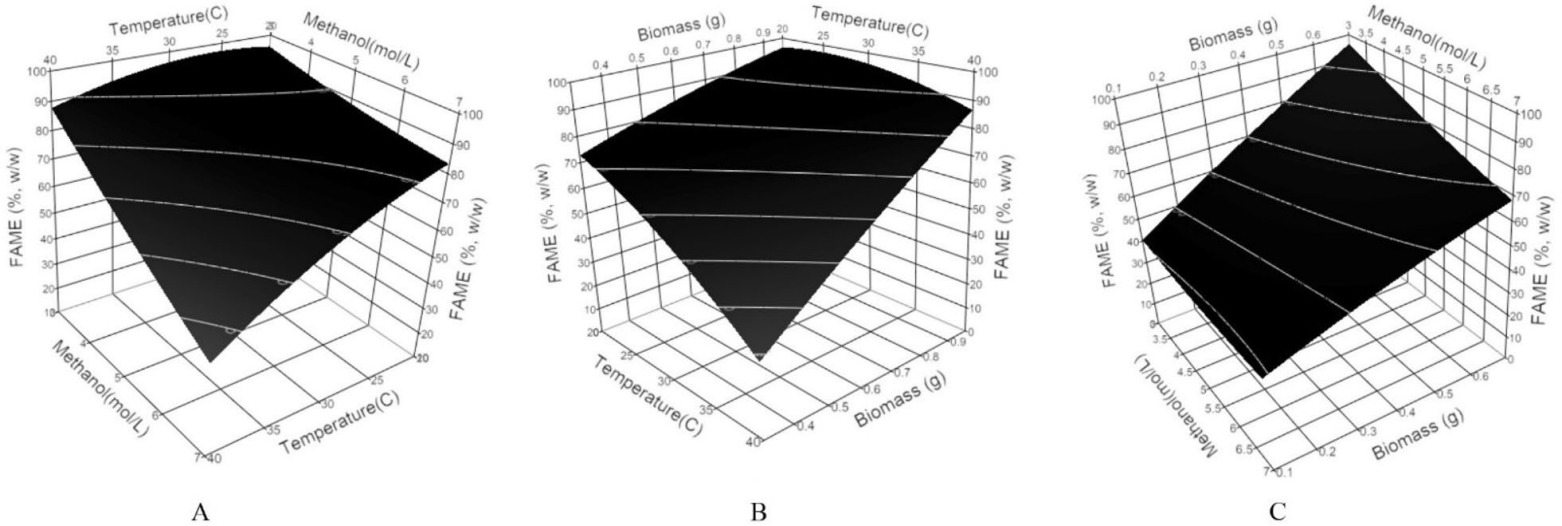
Response surface plots for FAME (%, w/w) showing the interactive effects. Hold values are **A** Biomass = 0.7 g, **B** Methanol = 5 mol/L, and **C** Temperature = 30 °C

Surface plots (Fig. 5) were constructed to determine the optimum conditions, where the response was plotted on the Z axis against the two of the investigated factors were plotted on the X and Y axes. In contrast, the third factor was set to the given value.

A significant linear correlation between the response and X_1_, X_2,_ and X_3_, where temperature and methanol concentration had negative estimates, while the amount of biomass used had a positive effect. The interaction influences of the investigated factors were significant, where X_1_X_2_ and X_2_X_3_ had negative estimates, while X_1_X_3_ had a positive estimate. The model was set to maximize the FAME concentration, and the suggested optimum conditions were X_1_ = 25.5 °C, X_2_ = 3 mol/L, and X_3_ = 0.7 g. A FAME concentration of 97.5 ± 8.91%, w/w, was predicted under the suggested conditions. A validation experiment was conducted where the actual FAME concentration produced was 95.53 ± 1.59%, w/w.

## Discussion

In the transesterification reaction, the inhibitory effect of methanol as an acyl-acceptor on the catalytic enzyme has been reported [11]. Several strategies have been tested to overcome the inhibitory effect of alcohols, such as using solvents along with the methanol that increases the reaction rate [12] and replacing methanol with methyl or ethyl acetate [13]. Stepwise addition [14] and replacing methanol with longer-chain alcohols (Mateos et al., 202) were adopted during this study.

Fungal biomasses of *A. flavus* and *R. stolonifer* could not catalyze the production of FAAE, where alcohols were added in a single dose from time zero. Isopropanol was tested as an example of secondary alcohol acyl-acceptors. Isopropanol inhibited the transesterification of TG, where *R. stolonifer* biomass was used as the catalyst, while a relatively low FAAE concentration was achieved where *A. flavus* biomass was employed, indicating the inhibitory effect of secondary alcohol isopropanol, which might result from the competitive inhibition effect of secondary alcohol [16].

On the other hand, a clear trend was observed when *A. flavus* biomass was used. The longer the alcohol chain, the less the FAAE produced; this trend was also observed when some lipases, such as Novozym SP435 in an n-hexane medium and lipase SP-35 in a solvent-free medium were tested to produce FAAE [17, 18]. FAAE maximum concentration was detected in reactions where methanol was used. Hence methanol was selected for further investigations. The strains used in this study were isolated and mutated in previous studies [19, 20], where the screening and selection of potent catalytic biomasses relied on using methanol as an acyl-acceptor which might create a positive bias towards methanol. However, methanol is still a vital cheap option readily available and recoverable.

A dominant Ping pong Bi Bi reaction mechanism is thought to be employed in the lipase-catalyzed transesterification reaction. TG or DG binds to the active site of the catalytic protein(s) embedded in the biomass, which catalyzes the separation of DG or MG and the formation of an intermediate protein-acyl complex intermediate. The protein-acyl complex intermediate bind to methanol, where the formation of FAME is catalyzed, and FAME and catalytic protein(s) are released. The Ping pong Bi Bi reaction mechanism has been reported for TG transesterification in a solvent-free medium using immobilized lipases and lipases of fungal origin [21, 22]. The accumulation of FFA, DG, and MG in reactions catalyzed by *R. stolonifera* might result from the slower reaction rate and/or competitive alcohol inhibition [23], which might not influence the *A. flavus* biomass catalytic activity. The difference between the catalytic behavior of the biomasses suggests that combining both biomasses in the same reaction vessel might bring further understanding. Thus, biomass mixtures were prepared, as shown in Table 4, and each mix was tested as a catalyst for TG transesterification.

Lipase cocktails or mixtures have been reported as an efficient technique to facilitate hydrolysis and transesterification [24, 25]. Different biomass mixtures were tested as catalysts for the transesterification of TG in WFO. The catalytic capability of mixtures that contain *R. stolonifer* biomass was significantly improved compared to pure *R. stolonifer* biomass, i.e., the more the proportion of *A. flavus* biomass, the higher the FAME concentration. Hence, *A. flavus* biomass was selected for further trials. However, no antagonistic effect between the biomasses was observed, and the potential of mixing two fungal biomasses or even more is an option. In other words, biomasses cocktails might be a legitimate possibility as the enzyme's cocktails, which might provide a cheaper technical solution for industrial applications, but still require further investigation.

Using microalgae for wastewater treatment provides a sustainable alternative to traditional wastewater treatment processes. Microalgae biorefineries allow inexpensive and energy-saving wastewater treatment and the recovery of add-value compounds [26]. *Chlorella sorokiniana* is a well-studied candidate for wastewater treatment [27–29]. The co-cultivation of fungi and microalgae has been studied lately for several purposes, including harvesting microalgae by flocculation [30, 31]. The pretreatment of algal feedstock has tentatively explored the ability of fungi to utilize algae as the sole source of nutrients [32–34].

The ability of *A. flavus* to grow efficiently on *C. sorokiniana* feedstock and the capability of the produced biomass to catalyze the transesterification reaction might provide an additional piece of the puzzle of biorefinery. The use of fungal biomass cultivated on the microalgae recovered from wastewater treatment for the catalysis of transesterification reaction, but this puzzle piece needs evaluation at a larger scale.

Reaction temperature, acyl-acceptor concentration, catalyst concentration, and reaction time have been reported to be the most crucial factors influencing transesterification reactions [35, 36]. One of the main drawbacks of biocatalytic production of FAME using a stepwise methanol addition strategy is the reaction time. In previous experiments, the reaction was carried out for 72 h, where tested acyl-acceptors were added on 24 h basis. Adding the final acyl-acceptor concentration at the beginning of the reaction inhibited the tested biomass's catalytic capability. Thus, the reaction time was fixed to 24 h, and methanol addition was carried out in four doses on a six hour basis for the following experiments. Temperature, Methanol concentration, and catalyst concentration were optimized using CCD. The adequacy of the model was verified, and the prediction formula was simplified. The high temperature and methanol concentration levels negatively influenced the catalyst capability, especially when combined with a low biomass concentration level, where the FAME concentration was dropped in runs 27 – 28 to 2.9%. The negative impact of temperature methanol interaction could be understood in light of the methanol's toxic influence on the catalytic proteins, which was reported to increase with higher temperatures [37, 38]. The optimum conditions validation experiment showed that the produced FAME concentration was in the predicted range considering the standard errors suggested by the model.

## Conclusion

Fungal biomasses of *A. flavus* and *R. stolonifer* showed considerable capability for catalysis of the transesterification of triglycerides in WFO. Single-dose addition of short-chain alcohols had a toxic effect on the fungal biomass catalytic capability. Thus stepwise addition of acyl-acceptor provided a proper alternative strategy with minimal toxicity. Isopropanol as branched alcohol reduced the transesterification capability of fungal biomass, while methanol was the most potent acyl-acceptor. The catalytic capability of *A. flavus* biomass was superior to *R. stolonifer,* and different accumulation rates of transesterification reaction intermediates accompanied the difference in activity. Biomass mixtures efficiently catalyze the transesterification reaction. However, the more the ratio of *A. flavus* biomass, the more efficient the catalytic capability suggesting that there is no antagonism when different biomasses are mixed. The biorefinery approach was valid as a remarkable biomass yield was produced when *A. flavus* was cultivated on the biomass of *C. sorokiniana* cultivated in synthetic wastewater. The produced biomass catalyzed the transesterification reaction with the same efficiency as *A. flavus* biomass produced on synthetic culture media. Statistical optimization of the transesterification reaction using *A. flavus* biomass as a catalyst improved the FAME produced to a final concentration of 95.53 ± 1.59%, w/w.

## Methods

### Microbial strains

In previous studies, several fungal isolates belonging to Aspergilli and Mucoralean fungi were screened for their biomasses' capability of catalyzing the methanolysis of WFO. Potent isolates were identified as *Aspergillus flavus* NDA04a (MK811208) and *Rhizopus stolonifer* 1aNRC11 (MN689079). Selected isolates were chemically mutated, and *A. flavus* NDA04a mutant D and *R. stolonifer* 1aNRC11 mutant G were used in this study [19, 20]. The mutant was preserved on potato dextrose agar (PDA) slants supplemented with olive oil, 1%. The inoculum was prepared by subculturing the mutant on PDA plates incubated at 28 °C for three days. Agar disks (5 mm diameter) were used as inoculum.

*Aspergillus flavus* NDA04a was cultivated in a control culture medium containing 5% glucose, 7% urea, 0.9% KH_2_PO_4_, 0.09% MgSO_4_.7H_2_O, and 3% olive oil, w/v, with an initial pH of 5. Sterile 50 mL cultures were inoculated with three disks (0.5 cm) and incubated in an orbital shaker at 28 °C and 200 RPM for four days.

*Chlorella sorokiniana* was kindly provided by the culture collection of Algae, City University of applied science, Bremen, Germany. Pre-cultures were cultivated axenically in Wuxal medium (a commercially available liquid plant fertilizer consisting of 8% N, 8% P_2_O_5_, 6% K_2_O, 0.01% B, 0.004% Cu, 0.02% Fe, 0.012% Mn and 0.004% Zn (Wilhelm Haug GmbH & Co.KG Germany)).

### Biorefinery approach

#### Production of *Chlorella sorokiniana* biomass

Synthetic wastewater was prepared according to the composition listed in Table 5 [39], 500 mL cultures were inoculated with 50 mL pre-cultures of *Chlorella sorokiniana* incubated at room temperature on an orbital shaker, and no additional light source was applied. Culture viability was observed using optical density at 680 nm. Once the optical density showed stable values, the culture pH was adjusted, then distributed to 50 mL cultures, supplemented with olive oil 3%, w/v, and autoclaved.

**Table 5:** Synthetic wastewater composition

Salt	Concentration (mgL^−1^)
K_2_HPO_4_	84
MgCl_2_	45
NH_4_Cl	350
CaCl_2_ ·2H_2_0	38
NaHCO_3_	47
C_10_H_14_N_2_Na_2_O_8_ ·2H_2_O(Na-EDTA)	280
Trace Metals Solution	1 mL/L
Trace Metals Solution (× 1000 Stock)	
MnCl_2_ ·4H_2_O	300
AlCl_3_ ·6H_2_O	1700
ZnSO_4_	200
Na_2_MoO_4_ ·2H_2_O	24
CoCl_2_ ·6H_2_O	12
CuSO_4_	20
FeSO_4_ ·7H_2_O	3000
C_10_H_14_N_2_Na_2_O_8_ ·2H_2_O(Na-EDTA)	5000

#### Production of fungal biomass

*A. flavus* NDA04a was cultivated on *C. sorokiniana* feedstock, where microalgal biomass was suspended in tap water at different concentrations and 3%, w/v, olive oil. Autoclaved cultures were inoculated with three disks (0.5 cm) and incubated in an orbital shaker at 28 °C and 200 RPM for four days.

*Rhizopus stolonifer* 1aNRC11 was cultivated in a control culture medium containing 5% glucose, 2.26% Fishmeal, 3% KH_2_PO_4_, 0.09% MgSO_4_.7H_2_O, and 3% olive oil, w/v, with an initial pH of 7.4. Sterile 50 mL cultures were inoculated with three disks (0.5 cm) and incubated in an orbital shaker at 25 °C and 200 RPM for three days.

Biomasses were collected by filtration using Whatman filter paper no. 1. Harvested biomass was washed thoroughly three times with tapwater, followed by lyophilization using a freeze dryer (Martin Christ, alpha LSC basic; Germany).

### Transesterification reaction

Fractured lyophilized fungal biomass, 0.5 g, was used as the biocatalyst for an emulsion of 5 g WFO and 0.75 mL of 1 M phosphate buffer pH 7.5 in a 50 mL Erlenmeyer flask. The reaction was carried out at 35 °C and 250 rpm for 72 h. Alcohol doses were added at 0, 24, and 48 h to a final concentration of 3 M. Methanol, Ethanol, Isopropanol, and Butanol were tested.

### Lipase assay

Lyophilized biomass (0.5 g) was inoculated to an emulsion of 5.5 g WFO and 30 mL of 1 M Tris buffer, pH 7.5. The reaction was carried out in an orbital shaker at 35 °C and 200 rpm for two h. The reaction mixture was centrifuged for 10 min at 5000 rpm. A gram of supernatant, two drops of phenolphthalein color indicator, and 25 mL diethyl ether and ethanol (1:1) solvent mixture were titrated against freshly prepared 0.1 N NaOH in 100 mL Erlenmeyer flask. Lipase activity (as the amount of enzyme required to produce 1 μmol free fatty acid per min) was determined in unit per gram cell weight (U/g) [40].

### Response surface methodology (RSM)

Reaction temperature, Methanol concentration, and biomass concentration were optimized using a central composite design (CCD). The investigated levels of each factor are presented in Table 6, and the response was the final FAME concentration. Reactions were carried out using different biomass weights to catalyze the transesterification of triglycerides in an emulsion of 5 g WFO and 0.75 mL of 1 M phosphate buffer pH 7.5 in a 50 mL Erlenmeyer flask. The reactions were carried out at different temperatures and 250 rpm for 24 h. Methanol doses were added to a final given methanol concentration at 0, 6, 12, and 18 h.

**Table 6:** Experimental levels of CCD factors

Symbol	Factor	Level		
		− 1	0	1
X_1_	Temperature (°C)	20	30	40
X_2_	Methanol concentration (mol/L)	3.0	5.0	7.0
X_3_	Biomass amount (g/5 g WFO)	0.1	0.4	0.7

### FAAE analysis

The reaction mixture was transferred to a 15 mL centrifuge tube and spun at 10,000 rpm for 5 min. Fatty acid methyl ester (FAME) was detected in the supernatant by thin-layer chromatography (TLC) with silica gel 60 F_254_ (E. Merck, Mumbai, India) using a solvent system of hexane /diethyl ether /acetic acid. Spots were stained in an iodine chamber and were investigated by Just TLC software (Sweday, Lund, Sweden).

Agilent Technologies 6890N gas chromatography (GC) provided a flame ionization detector, and a capillary column (HP-5 5% phenyl methyl siloxane, 30 m by 320 μm by 0.25 mm) was used to quantify the FAMEs content in the supernatant. Peaks determination was carried out by comparing the retention time of FAMEs of the sample (100 mg) and a known concentration of FAME standard mixture C8 – C24 (Sigma-Aldrich Chemical Co. St. Louis, MO, USA), each dissolved in 1 mL hexane. One μL sample was injected into the GC, where the oven was adjusted at 210 ℃, isothermally for 15 min, and helium was used as the carrier gas.

### Statistical analysis

The data obtained in triplicates were subjected to statistical analysis using IBM SPSS Statistics (Version 16. IBM, Chicago, USA), where one-way analysis of variance (ANOVA), followed by mean comparison using the LSD test, was carried out. Response surface methodology was designed and analyzed using JMP statistical software (Version 8. SAS Institute Inc., Cary, NC), where duplicates of each design run were conducted.

## Data Availability

The datasets generated and analyzed during the current study and not included in this published article are available from the corresponding author upon reasonable request.
